# Ion Transport in (Localized) High Concentration Electrolytes for Li-Based Batteries

**DOI:** 10.1021/acsenergylett.3c01662

**Published:** 2024-01-05

**Authors:** Helen K. Bergstrom, Bryan D. McCloskey

**Affiliations:** † Department of Chemical & Biomolecular Engineering, University of California, Berkeley, California 94720, United States; ‡ Energy Storage and Distributed Resources Division, Lawrence Berkeley National Laboratory, Berkeley, California 94720, United States

## Abstract

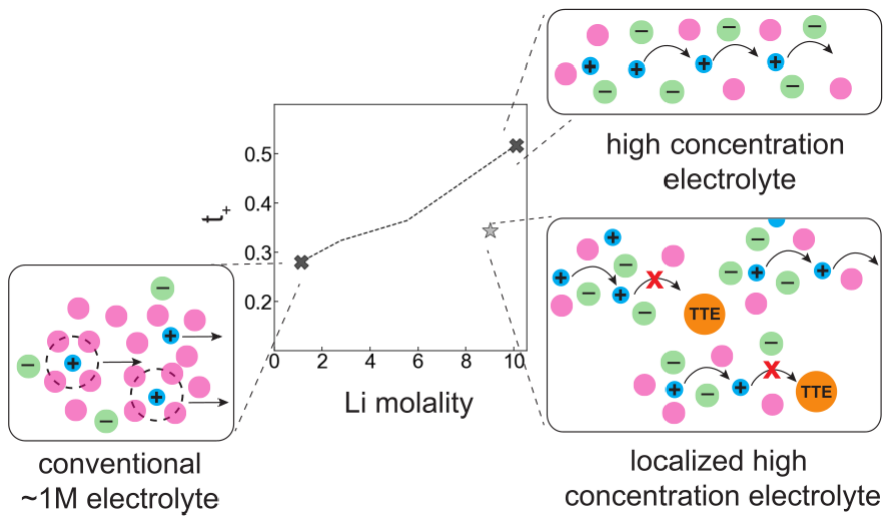

High concentration electrolytes (HCEs) and localized high concentration electrolytes (LHCEs) have emerged as promising candidates to enable higher energy density Li-ion batteries due to their advantageous interfacial properties that result from their unique solvent structures. Using electrophoretic NMR and electrochemical techniques, we characterize and report full transport properties, including the lithium transference numbers (*t*_+_) for electrolytes ranging from the conventional ∼1 M to HCE regimes as well as for LHCE systems. We find that compared to conventional electrolytes, *t*_+_ increases for HCEs; however the addition of diluents to LHCEs significantly decreases *t*_+_. Viscosity effects alone cannot explain this behavior. Using Onsager transport coefficients calculated from our experiments, we demonstrate that there is more positively correlated cation–cation motion in HCEs as well as fast cation–anion ligand exchange consistent with a concerted ion-hopping mechanism. The addition of diluents to LHCEs results in more anticorrelated motion indicating a disruption of concerted cation-hopping leading to low *t*_+_ in LHCEs.

High concentration electrolytes (HCEs) have gained significant attention over the past decade for their lower flammability, advantageous interfacial properties, and promise of enabling lithium metal anodes and high-voltage cathodes.^1−5^ While promising from an interfacial standpoint, HCEs are more expensive and have lower conductivity and have significantly higher viscosity than traditional ∼1 M electrolytes. Recently, localized high concentration electrolytes (LHCEs), where an inert nonsolvating but miscible diluent is added to high concentration electrolytes, have been proposed as a lower cost alternative to HCEs that still possesses favorable interfacial properties.^6−8^

Concentrated electrolytes (≳0.1M) can generally be broken into three regimes: salt-in-solvent electrolytes where there is more solvent than needed to fill the cation’s primary solvation shell, salt-solvate electrolytes or solvate ionic liquids where the number of solvent molecules is sufficient to fill the primary solvation shell without excess free solvent, and solvent-in-salt electrolytes where there are insufficient solvent molecules to fill the primary solvent shell of the cation.^5^ The superior interfacial properties of solvent-in-salt electrolytes are attributed to the unique solvent structure resulting from lack of uncoordinated solvent molecules and participation of anions in cation solvation which leads to preferential anion reduction to form the solid-electrolyte interphase (SEI), as well as increased oxidative stability at the cathode.^9−11^

The vastly different solvation environment in solvent-in-salt electrolytes that leads to improved interfacial properties is likely to result in different transport phenomena. The dearth of coordinating solvents at high concentrations is believed to result in networks of lithium–anion–lithium coordination sites.^11^ This network is hypothesized to lead to structural (hopping) lithium transport as opposed to more vehicular–solvent coordinated motion seen at low concentrations.^10,12−14^ It should be noted that in highly concentrated systems, the salt and solvents have similar volume fractions and that during cell polarization, solute-volume effect-driven transport (e.g., Faradaic convection) becomes a relevant transport mechanism in addition to electric-field-driven transport and concentration-gradient-driven diffusive transport.^15−18^ Despite strong evidence of distinctive ion-coordination networks,^12−14^ there are relatively few studies focusing on measuring transport properties in HCEs and LHCEs beyond conductivity. Quantifying and characterizing transport properties and Li-transport mechanisms in HCEs and LHCEs are essential to understanding the interplay and trade-offs between interfacial properties and bulk transport properties that have broad implications for rate-performance, efficiency of charging, and battery safety. With the exception of recent work by Wang et al., existing transport property studies almost entirely rely on self-diffusion coefficients or Bruce–Vincent type measurements which require ideal solution assumptions and therefore are not capable of giving rigorous insight into the roles of ion–ion and solvent–ion correlations.^15^

To fill this gap in understanding, using electrophoretic NMR (eNMR) and electrochemical techniques, we rigorously experimentally quantify the full transport properties of ions and solvents in lithium bis(fluorosulfonyl)imide (LiFSI) in dimethyl carbonate (DMC) at concentrations ranging from the typical salt-in-solvent regime to the saturated-solvent-in-salt HCE regime. We also study LHCE systems composed of LiFSI in DMC with 1,1,2,2-tetrafluoroethyl-2,2,3,3-tetrafluoropropyl ether (TTE) added as a diluent. Table 1 summarizes the electrolytes explored in this study, with preparation and characterization fully described in the Supporting Information. First, we present an analysis of molar conductivity, self-diffusion coefficients, and the total salt diffusion coefficient as a function of salt concentration and their relationship to solution viscosity. Similar to previous studies, we find that viscosity effects alone are insufficient to describe transport property differences across the classes of studied electrolytes.^4,16,19,20^ Next we examine the electrophoretic mobility and transference number. Without making any assumptions about solution ideality, we demonstrate through Onsager transport theory that HCEs have high transference numbers owing to a decrease in cation–anion correlated motion and an increase in positive cation–cation correlations, consistent with a coordinated Li-ion hopping transport mechanism. We find that LHCEs do not experience an improvement in transference number and experience more negative cation–cation correlated motion, indicating either a change in the transport mechanism or ion-dissociation with the addition of diluent.

**Table 1:** Solution Properties for High Concentration and Localized High Concentration Electrolytes

molality *m*	molar ratio	wt. %	particle	density	molarity M	viscosity
(mol kg^–1^ DMC)	(LiFSI:DMC:TTE)	salt	fraction	(g mL^–1^)	(mol L^–1^)	(mPa·s)
1.1	1:10:0	17.19	0.0833	1.176	1.08	1.81 ± 0.04
2.78	1:4.0:0	34.21	0.1667	1.304	2.38	6.56 ± 0.06
5.55	1:2.0:0	50.94	0.2500	1.431	3.90	34.79 ± 0.11
10.11	1:1.1:0	65.41	0.3228	1.571	5.41	248.89 ± 1.20
9.0	1:1.23:0.62	42.39	0.2597	1.545	3.49	45.55 ± 0.21
5.55	1:2.0:1	31.21	0.2000	1.474	2.46	10.97 ± 0.07

**Viscosity vs Solvation Effects.** As expected, conductivity is significantly reduced in high concentration electrolytes dropping an order of magnitude from 10 mS/cm for the 1.1 *m* salt-in-solvent electrolyte to 1.2 mS/cm for the 10.11 *m* solvent-in-salt electrolyte (see Figure 1a). This decrease in conductivity corresponds with a >100-fold increase in viscosity; however, reduction in conductivity cannot be attributed to viscosity alone. Examining the 9.0 *m* LHCE, there is a 80% decrease in solution viscosity yet only a 20% increase in electrolyte conductivity in comparison to the corresponding HCE. For the 5.55 *m* LHCE both solution viscosity and conductivity decrease. For a dilute colloidal solution, the Stokes–Einstein relationship dictates that the diffusion coefficient of species *i* is related to the solution viscosity according to
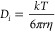
1where η is the solution viscosity and *r* is the particle radius or ionic radius for electrolytes.^21^ Combining this relationship with the Nernst–Einstein relationship yields Walden’s rule which dictates that the product of molar conductivity (Λ) and viscosity is constant according to^22^
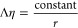
2Applying Walden’s analysis to the LiFSI in DMC systems, in line with previous works, in Figure 1b we see clearly that the product Λη is not constant across salt concentration or with the addition of diluent.^4,16,19,20^ This indicates that more complex ion and solvent interactions are responsible for the decrease in conductivity at low solvent:salt ratios (high concentrations). If we were to assume only ideal interactions and full ion dissociation, this would suggest that the effective ionic radius decreases with an increasing salt concentration.

**Figure 1:**
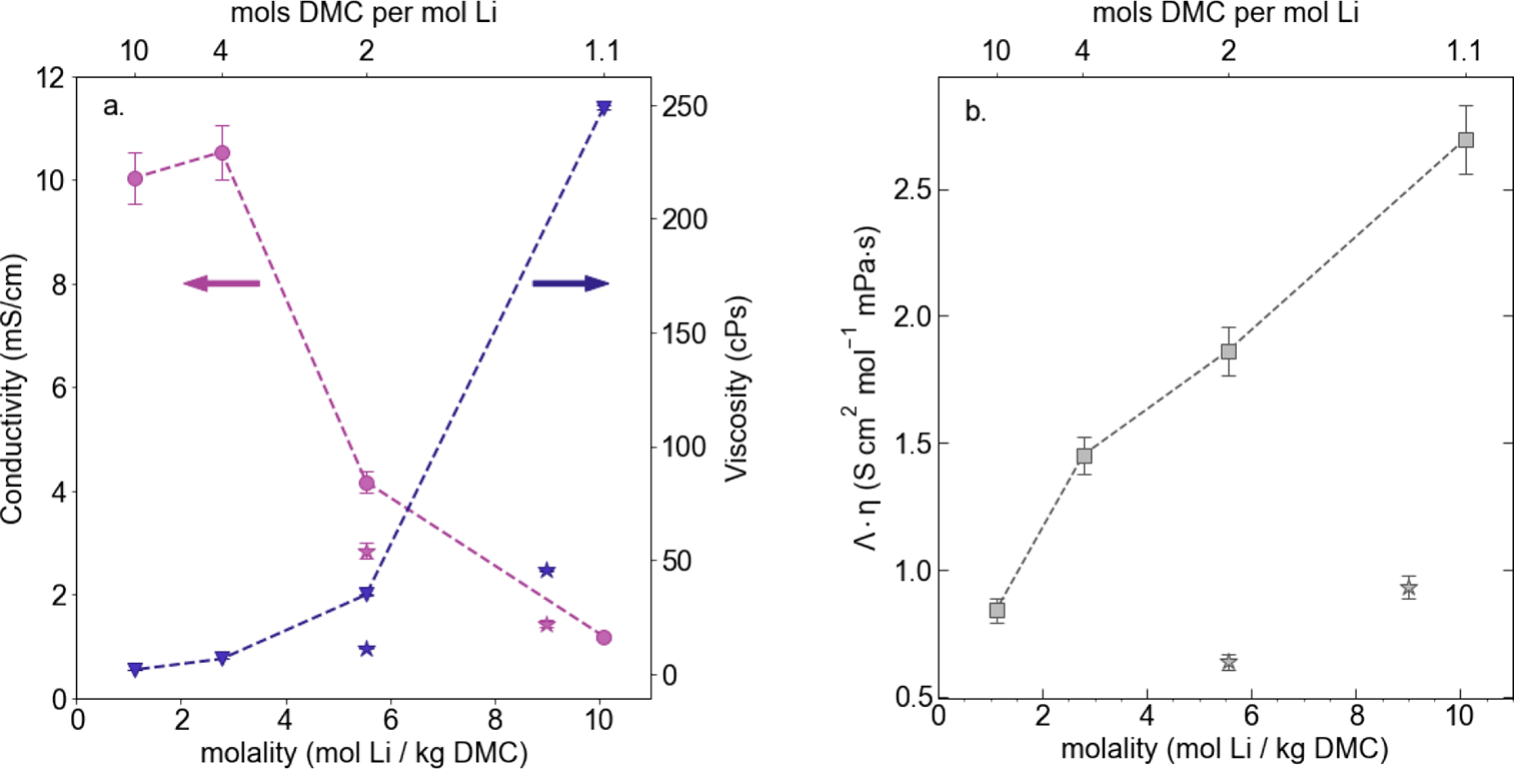
(a) Conductivity (mS/cm) and viscosity (cP s) vs molality (mol Li^+^/kg DMC) and DMC:Li molar ratio. (b) Product of molar conductivity and viscosity (S cm^2^ mol^–1^ mPa·s) vs molality (mol Li^+^/kg DMC) and DMC:Li molar ratio. Star symbols denote LHCE systems composed of LiFSI in DMC and TTE. Molalities are reported with respect to DMC weight and not total solvent (DMC + TTE) weight.

Conductivity data suggest overall slowing ion motion with increased salt concentration but does not give any species-specific insight. Self-diffusion coefficients of each species (*D*_*i*_^self^), as measured using pulsed-field gradient (PFG) NMR, are reported in Figure 2a. As expected from the increase in viscosity with increasing molality (i.e., decreasing solvent to salt ratio), *D*_*i*_^self^ decreases for all species as molality increases. In both LCHE systems, the TTE self-diffusion coefficient is significantly higher than that of either ion or the DMC, an indication that the TTE is not part of the primary ion solvation sheath.^8^ This is in agreement with solvent structures obtained from ab initio molecular dynamics and Raman measurements.^6,23^ Examining the product of self-diffusion coefficients and viscosity, *ηD*_*i*_^self^ is relatively constant for DMC molecules across the entire studied range of salt concentration, suggesting solvent self-diffusion can largely be explained by changes in solution viscosity (see Figure 2b). We do observe a slight decrease in *ηD*_0_^self^ as salt concentration increases from 1.1 *m* to 2.78 *m* to 5.55 *m*, corresponding to decreasing amounts of free solvent. Surprisingly, we see a small increase in η*D*_0_^self^ for the 10.1 *m* HCE, where Raman measurements indicate there is no free solvent, compared to 5.55 *m* where a small fraction of free solvent exists.^6^ This could indicate that the DMC–Li interactions are weaker or there is faster ligand exchange at 10.1 *m* than at 5.55 *m*.

**Figure 2:**
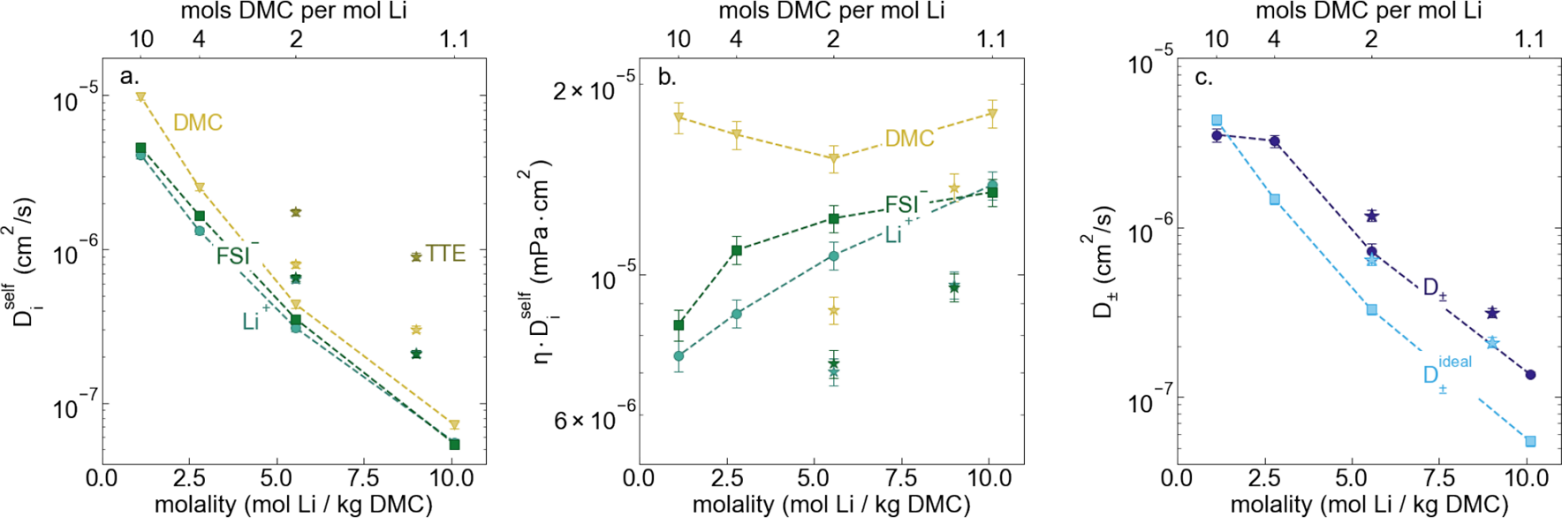
(a) Self-diffusion coefficients (cm^2^/s) vs molality (mol Li^+^/kg DMC) and DMC:Li molar ratio, as measured using PFG NMR, and (b) product of self-diffusion coefficient and viscosity (mPa cm^2^) vs molality (mol Li^+^/kg DMC) and DMC:Li molar ratio. (c) Total salt diffusion coefficients (cm^2^/s) vs molality (mol Li^+^/kg DMC) and DMC:Li molar ratio as measured using restricted diffusion and as calculated from PFG NMR results presented in panel a, assuming ideal solution behavior. Star symbols denote LHCE systems composed of LiFSI in DMC and TTE. Molalities are reported with respect to DMC weight and not total solvent (DMC + TTE) weight.

We observe that η*D*_*i*_^self^ is not constant for the Li^+^ ion and FSI^–^ ions and instead increases for both ions as the concentration increases. Self-diffusion coefficients are measures of the rate of ideal Brownian motion. Nonideal interactions such as ion pairing should not explicitly affect *D*_*i*_^self^; therefore, the change in η*D*_*i*_^self^ with concentration would suggest a change in the effective ion radius in the different solvation environments with increasing concentration, resulting in larger ion radii. Interestingly, η*D*_*i*_^self^ is significantly smaller for DMC, Li^+^, and FSI^–^ in the 1.23:0.62:1 DMC:TTE:LiFSI LHCE compared to its HCE counterpart, which could indicate that the TTE diluent has an effect on the strength of ion–ion and ion–solvent interactions. Measurements of LiFSI:DMC systems diluted with bis(2,2,2-trifluoroethyl) ether (BTFE) showed a downshift in FSI^–^ Raman band corresponding to a slight weakening in the association of Li^+^ and FSI^–^1.^6^ Previous studies of sulfolane-based LHCEs also report this effect which they attribute to diluent driven ion-dissociation due to the significantly lower dielectric constant of diluents compared to sulfolane.^19,24^ In the LHCE systems studied here, dielectric properties are unlikely to explain the change in solvation environment upon TTE addition, as TTE and DMC have similar dielectric constants (6.2 and 3.2, respectively).^25,26^

Next we consider the total salt diffusion coefficient (*D*_±_) as measured by restricted diffusion. Again we observe *D*_±_ decreases rapidly with increasing salt concentration in the highly concentrated regime. However, unlike the self-diffusion coefficients, *D*_±_ is approximately constant at ∼3 × 10^–6^ cm^2^/s between the salt-in-solvent and salt-solvate regime, despite the 4:1 DMC:Li (2.8 *m*) electrolyte having a viscosity ∼3.5 times larger than the 10:1 DMC:Li (1.1 *m*) salt-in-solvent electrolyte. This is not true for the ideal solution total salt diffusion coefficient (*D*_±_^ideal^) calculated from self-diffusion coefficients using the Nernst–Hartley relationship and the data shown in Figure 2a.^27^ Comparing *D*_±_ to *D*_±_^ideal^, we see that for both solvent-in-salt and salt-solvate electrolytes *D*_±_ is larger than *D*_±_^ideal^ (see Figure 2c). The change in the ratio of *D*_±_ to *D*_±_^ideal^ with salt concentration can primarily be attributed to changes in the thermodynamic factor (χ) for all electrolytes except the 1.1:1 DMC:Li (10.1 *m*) HCE for which the thermodynamic factor alone cannot explain this behavior (see Supporting InformationFigure S4). After accounting for changes in thermodynamic factor, *D*_±_/*D*_±_^ideal^ ≈ 2 is indicative of an increase in positive distinct ion-correlations for the 1.1:1 DMC:Li electrolyte which speed up overall salt transport. This speed-up behavior has previously been observed in ligand functionalized polymer membranes and polyelectrolyte solutions.^28,29^

From eNMR data, we directly obtain electrophoretic mobilities (μ_*i*_) of the ions and solvent species with reference to a stationary frame. We note that to suppress convection in the lowest viscosity samples, 3–4 wt % PVDF was added as a gelling agent to the 1:10 DMC:Li (1.1 *m*) and 1:4 DMC:Li (2.78 *m*) electrolytes (see Supporting Information section S1.7). As salt concentration increases, we see a decrease in electrophoretic mobility for all species, with the FSI^–^ ion experiencing the greatest overall decrease in mobility with increasing concentration (see Figure 3a). The positive and significant electrophoretic mobility of DMC across all concentrations is notable particularly for the solvent-in-salt electrolytes where μ_+_ ≈ μ_0_. Often solvent motion in the same direction as Li^+^ under an electric field is attributed to a vehicular Li^+^ transport mechanism; however momentum and local volume conservation^30−32^ in these systems dictate that solvent transport should move in the opposite direction as the anion to counteract the motion of the anion which makes up a significantly larger mass fraction than the lithium cation. Therefore, from these macroscopic mobility measurements alone, we cannot make any molecular-level conclusions as to the Li^+^ transport mechanism (i.e., structural or vehicular motion).

**Figure 3:**
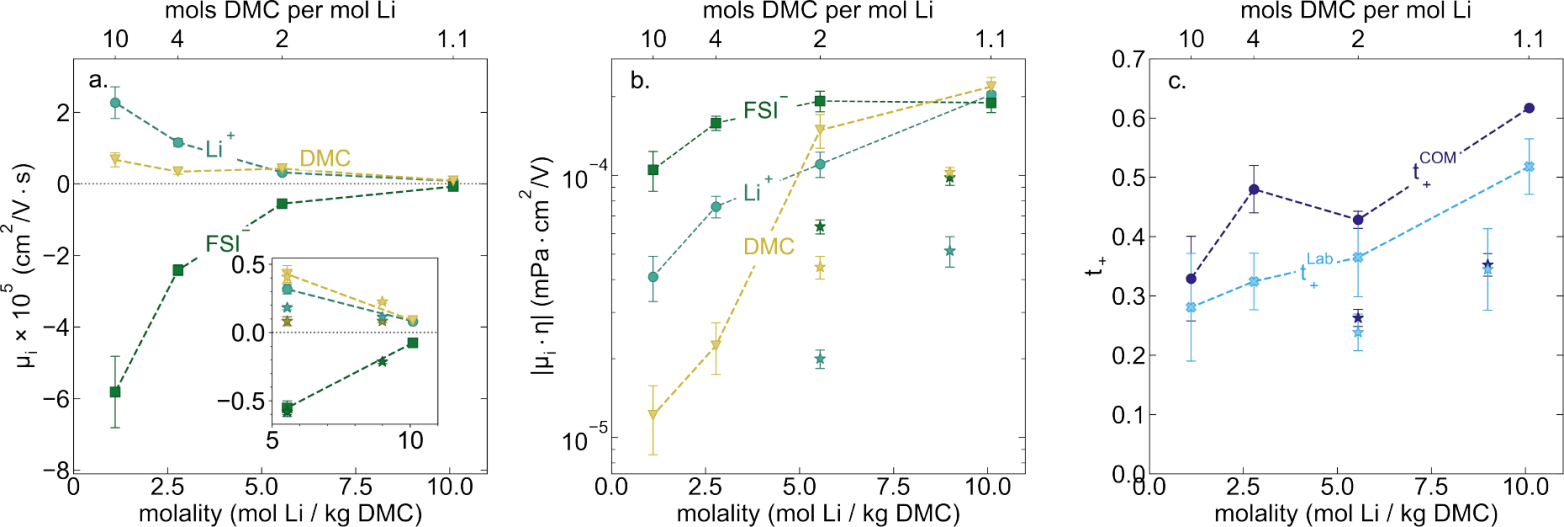
(a) Electrophoretic mobilities (cm^2^/(V·s)) vs molality (mol Li^+^/kg DMC) and DMC:Li molar ratio measured by eNMR. (b) Viscosity normalized electrophoretic mobilities (mPa cm^2^/V) vs molality (mol Li^+^/kg DMC) and DMC:Li molar ratio. (c) Li^+^ transference number vs molality (mol Li^+^/kg DMC) and DMC:Li molar ratio as measured by eNMR. Star symbols denote LHCE systems composed of LiFSI in DMC and TTE. To reduce convective artifacts in eNMR measurements, it was necessary to add 3–4 wt % PVDF to the 1.1 *m* and 2.8 *m* LiFSI in DMC HCEs. The gel network equally impacts all species (Supporting Information section S1.7).

If we normalize electrophoretic mobilites accounting for viscosity by examining the product |μ_*i*_η|, we observe that |μ_–_η| increases only slightly with increasing concentration indicating that the FSI^–^ ion mobility is largely controlled by solution viscosity (see Figure 3b). Given the strong coordination between the Li^+^ and FSI^–^ ion in HCEs, this would require that ligand exchange occurs rapidly such that the FSI^–^ is still relatively mobile under an electric field. |μ_+_η| and |μ_0_η| both increase significantly with increasing salt concentration, again indicating that Li^+^ transport is strongly influenced by the solvation environment and not simply viscosity. |μ_*i*_η| is significantly decreased for all species in the LHCE systems compared to their corresponding HCEs which could be indicative of a change in strength of ion–ion and ion–solvent interaction as well as conduction mechanism.

**Transference Number and Ion-Correlation.** Using eNMR data, we can directly calculate the *t*_+_ according to eq 3, which can be derived from concentrated solution theory or the Onsager framework.^30−33^
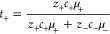
3*t*_+_ inherently requires the definition of a reference frame. Equation 3 is valid for the laboratory, center-of-mass, and solvent reference frames given the mobilities are calculated relative to that frame (see Supporting Information section S1.7). Herein we present the transference number from the laboratory frame (*t*_+_^Lab^) and the center-of-mass frame (t_+_^COM^). At low solvent-to-salt ratios the solvent reference frame is not particularly meaningful (for *t*_+_^0^ see Supporting InformationFigure S6), while *t*_+_^Lab^ and t_+_^COM^ are more accurate representations of the interpretation of lithium transference number as the fraction of the total current carried by Li^+^ ion under conditions of no concentration gradients.^31,32^*t*_+_^Lab^ and t_+_^COM^ both are highest at a 1.1:1 DMC:LiFSI ratio (10.1 *m*), reaching exceptional values of 0.52 and 0.62, respectively. With increasing solvent concentration (decreasing salt molality), *t*_+_^Lab^ decreases to ∼0.3 which is typical of salt-in-solvent electrolytes (see Figure 3c). There does not appear to be a step transition from the solvent-in-salt to solvate-salt or to the salt-in-solvent regimes as *t*_+_^Lab^ is reduced to ∼0.35 even for the 2:1 DMC:LiFSI (5.55 *m*) HCE.

Notably, *t*_+_ values in both the 1.23:0.62:1 DMC:TTE:LiFSI LHCE and 1:2:1 DMC:TTE:LiFSI LHCE are significantly smaller than their HCE counterparts in both the lab and center of mass reference frames. This is consistent with Bruce–Vincent-based current ratio measurements of sulfolane-based LHCEs^19^ and suggests that while LHCEs may improve conductivity and lower viscosity, it comes at the cost of lower transference number. This could also suggest that the addition of diluents in LHCE systems has an effect on the lithium conduction mechanism.

To gain insight on the transport mechanism, we can quantify the contributions of different ion-correlations to the overall solution conductivity using Onsager transport coefficients, *L*^*ij*^ calculated from experimental properties.^30^ For the binary LiFSI in DMC electrolytes, there are 3 independent Onsager coefficients: *L*^+ –^, *L*^++^, and *L*^– –^. *L*^+ –^ captures the correlated motion between the cation and anions, while *L*^++^ and *L*^– –^ capture the correlated motion between like charged particles. While not independent transport parameters, we can also calculate *L*^+0^ and *L*^–0^ which capture the cation–solvent and anion–solvent correlated motion, respectively. We note that while a ternary system should have 6 independent Onsager coefficients, in the LHCE systems of LiFSI in DMC and TTE, TTE is theorized to be locally phase-separated and therefore not a true single-phase ternary system. For simplicity’s sake, herein, we treat LHCEs as a binary system in our Onsager analysis which is equivalent to the assumption of a “mean” solvent. To simplify interpretation across the large range of concentrations studied, here we normalize *L*^*ij*^ by the molar electrolyte concentration in order to yield “per-ion” transport coefficients.

First we consider *L*^+ –^/*c*, the per-ion cation–anion correlation. *L*^+ –^/*c* is largest for the low concentration salt-in-solvent electrolyte and steadily decreases with increasing salt concentration (see Figure 4a). This behavior is counter to our typical understanding of ion pairing, where we would expect a higher degree of salt dissociation and therefore less cation–anion correlation at low salt concentration and more ion-pairing and aggregation at high salt concentration. However, a static picture of ion-pairing is not sufficient for understanding the correlated motion. One plausible explanation for less cation–anion correlation at low solvent:salt ratios is that while there are overall more ion pairs the residence time of these ion-pairs is shorter.^29,34^ This would imply that Li^+^ does not travel as far with its coordinating anions at high concentration, a phenomenon consistent with fast ligand exchange and structural Li^+^ motion. Similar behavior of decreasing *L*^+ –^ with increasing salt concentration have been seen in glyme-based salt-solvate electrolytes and sulfolane-based HCEs though we note that these studies used ideal transference number measurements for calculation of L^*ij*^ coefficients.^35,36^ In the 9.0 *m* LHCE system, *L*^+ –^/*c* is of similar magnitude to the corresponding HCE systems. This indicates that despite the slight change in ion-dissociation behavior seen with addition of TTE, the TTE diluent does not significantly effect cation–anion correlated motion in this system. However, in the 5.55 *m* LHCE system *L*^+ –^/*c* is double that of the corresponding HCE system, indicating that the TTE diluent increases cation–anion correlation. This would be consistent with a slowing down of ligand exchange at higher relative diluent concentrations.

**Figure 4:**
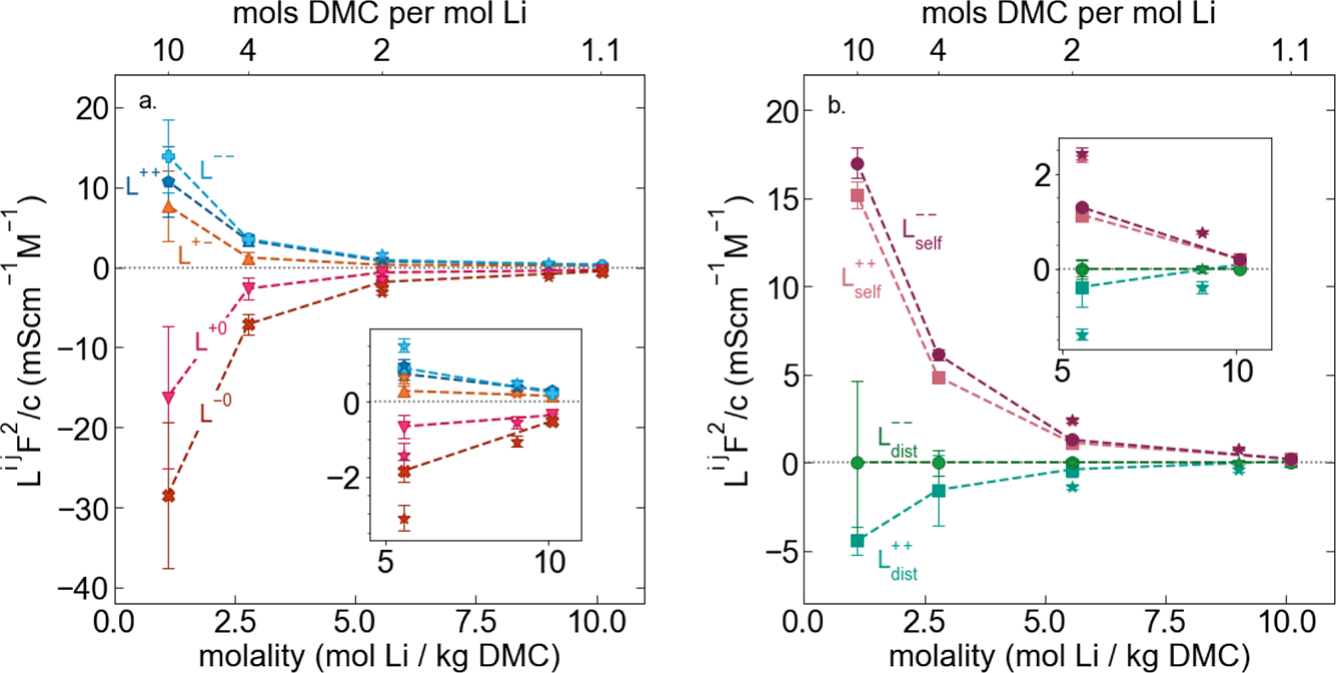
(a) Per ion Onsager transport coefficients calculated from experimental data. (b) Per ion Onsager transport coefficients *L*^++^ and *L*^– –^ broken into ideal (self) and distinct terms. Star symbols denote LHCE systems composed of LiFSI in DMC and TTE.

The transport coefficients *L*^*ii*^ can be separated into a self-term *L*_self_^*ii*^ which accounts for ideal self-diffusion, and a distinct term *L*_dist_^*ii*^ which captures correlations between particles.^30,37−40^ Directly corresponding to a decrease in *D*_*i*_^self^, *L*_self_^++^/*c*, and *L*_self_^– –^/*c*, both steadily decrease with increasing salt concentration (see Figure 4b). *L*_dist_^– –^ ≈ 0 across all studied concentrations, indicating that there is very little correlated anion motion, regardless of the solvation environment. *L*_dist_^++^ is highly negative at low salt concentrations, indicating significant cation–cation repulsion, which is consistent with the higher degree of ion dissociation. For the solvent-in-salt and salt-solvate HCEs *L*_dist_^++^ is ∼0 within error. While it is tempting to attribute this behavior to increased ion-paring and therefore lower cation effective charge at high concentrations, again, this static picture is not appropriate. In fact we observe that Li^+^ has a higher effective charge in the HCE regime than in the salt-in-solvent regime (see Supporting Information section S3). Using molecular dynamics, Yamada et al. saw in a 1.1:1 DMC:LiFSI HCE on average each FSI^–^ coordinated 2–3 Li^+^ which could plausibly lead to correlated motion of larger ion aggregates with multiple lithium ions coordinated to the same anion and therefore a less negative *L*_dist_^++^.^2^ However, given the low degree of cation–anion correlation in the solvent-in-salt systems, it is unlikely that there is significant vehicular motion of larger aggregates. Instead, *L*_dist_^++^/*c* ∼ 0 is more consistent with a concerted hopping mechanism for Li^+^ transport in the HCE systems in which the hopping of one Li^+^ into a new coordinating site pushes the Li^+^ previously occupying that environment to the next coordination site. Recent studies have suggested that diluents could interrupt percolated three-dimensional solvation networks effectively blocking Li^+^ hopping.^11,19,41^ Examining both LHCE systems, *L*_dist_^++^/*c* is significantly more negative than corresponding HCEs which is consistent with a decrease in positive cation–cation correlation that could arise from concerted hopping.

Finally, we can examine *L*^*i*0^ to look at solvent–ion correlations though again we note that *L*^*i*0^ are not independent transport parameters. *L*^+0^ and *L*^–0^ are both negative across all concentrations which is consistent with conservation of momentum.^30^ That *L*^+0^ is always less negative than *L*^–0^ reflects the fact that there is a more positive correlation between the Li^+^ and DMC molecules than the FSI^–^ and DMC.

In addition to interpretation of the Onsager coefficients, ion transport can also be examined through the analogous Stefan–Maxwell transport framework’s Stefan–Maxwell diffusivites  and their corresponding friction coefficients *K*_*ij*_ which are presented in the Supporting Information (see Supporting InformationFigure S7). We note that to be consistent with most literature, Stefan–Maxwell coefficients are calculated according to eqs S11–S14 using the transference number defined with respect to the solvent reference frame as opposed to the center of mass reference frame used for Onsager coefficients *L*^*ij*^. We observe that *K*_+ –_, which captures cation–anion drag interactions, increases with increasing salt concentration in the high-concentration regime in agreement with the previous studies of concentrated lithium hexafluorophosphate in ethyl methyl carbonate electrolytes.^15^ goes through a maxima around 2.8 *m* (4:1 DMC:LiFSI) corresponding to a peak in conductivity, a phenomena observed in propylene carbonate,^17^ ethylene carbonate,^15^ and fluorinated solvent^42^-based systems. In the solvent-in-salt regime, *K*_+ –_ and *K*_0+_ are the same order of magnitude, indicating that the solvent molecules and anion have similar strength pairwise frictional drag interactions with the lithium ion. This is explained by the necessity of anion participation in lithium solvation at high concentrations and is consistent with structural diffusion. *K*_0–_, which captures the frictional interactions between the solvent and anion, is at least an order of magnitude smaller than *K*_+ –_ across all concentrations and an order of magnitude smaller than *K*_0+_ for all concentrations except the 1 M electrolyte where they are the same order of magnitude. This is not entirely surprising, as lithium–solvent interactions are known to be significantly stronger than anion–solvent interactions. Most notably, *K*_0–_ transitions from positive to negative between 4 mol DMC per lithium and 2 mol DMC per lithium as the solvation shell is fully filled. This corresponds directly to solvent reference frame transference number transitioning from positive to negative (see Supporting InformationFigure S6).

**Summary and Outlook.** Complete transport and thermodynamic properties were rigorously measured for LiFSI in DMC electrolytes ranging from the salt-in-solvent to solvent-in-salt regimes as well as for localized high concentration electrolytes containing TTE as a diluent (Table 2). There are both sharp drops in conductivity and in ion self-diffusion coefficients with increasing salt concentration as we change solvation regimes from salt-in-solvent to salt-solvate to solvent-in-salt. These changes in conductivity and self-diffusion cannot be attributed to increasing viscosity effects alone and are indicative of changes in the effective Li-ion radius or in transport mechanism. By direct measurement of ion electrophoretic mobilities via eNMR, we calculate the true transference number of these systems and demonstrate that *t*_+_ increases with an increasing salt concentration. Notably we find that the 1.1:1 DMC:LiFSI (10.1 *m*) HCE has an exceptional transference number of 0.52. However, upon addition of TTE as a diluent, *t*_+_ drops to 0.35 for a 1.23:0.62:1 DMC:TTE:LiFSI (9 *m*) LHCE, and further increasing relative TTE concentration decreases Li transference with *t*_+_ dropping to 0.24 for the 2:1:1 DMC:TTE:LiFSI (5.55 *m*) electrolyte. This indicates that diluents in LHCEs are not truly inert and instead have an effect on the solvation environment and transport mechanism resulting in low transference numbers. By examining Onsager transport coefficients, we conclude that there is no significant vehicular motion of anion–cation aggregates in HCEs. We find that despite increased ion-dissociation, traditional salt-in-solvent electrolytes show the largest degree of cation–anion correlated motion that is indicative of longer-lasting ion pairs which decrease the lithium transference number. The small degree of cation–anion coordination in HCEs and LHCEs suggests rapid ligand exchange. We also observe that distinct cations have less anticorrelated motion at higher salt concentrations, a finding that is consistent with a concerted ion-hopping mechanism. However, we find that LHCEs have more anticorrelated cation–cation motion than their HCE counterparts, indicating that diluents are likely interrupting the cation-hopping mechanism. These findings are consistent with previous molecular dynamic studies and conclusions based on self-diffusion coefficients.^19^

**Table 2:** Transport and Thermodynamic Properties for High Concentration and Localized High Concentration Electrolytes

electrolyte	molar ratio	molarity	conductivity	*D*_±_ × 10^7^	*t*_+_^Lab^	χ
type	(LiFSI:DMC:TTE)	(mol L^–1^)	(mS/cm)	(cm^2^/s)		
solvent-in-salt	1:1.1:0	5.49	1.19 ± 0.06	1.36 ± 0.04	0.52 ± 0.05	1.16 ± 0.15
HCE	1:2.0:0	3.90	4.17 ± 0.21	7.23 ± 0.84	0.37 ± 0.07	2.38 ± 0.58
salt-solvate	1:4.0:0	2.38	10.54 ± 0.53	32.5 ± 2.7	0.32 ± 0.05	2.60 ± 0.69
salt-in-solvent	1:10:0	1.08	10.04 ± 0.50	35.3 ± 3.2	0.28 ± 0.09	0.67 ± 0.29
solvent-in-salt	1:1.23:0.62	3.50	1.43 ± 0.07	3.14 ± 0.18	0.35 ± 0.07	1.68 ± 0.35
LHCE	1:2.0:1	2.46	2.85 ± 0.14	11.8 ± 0.91	0.24 ± 0.03	2.42 ± 0.40

While it is clear that high concentration electrolytes have an improved transference number, this comes at the expense of an order of magnitude lower conductivity and diffusion coefficients. Addition of a diluent to LHCEs, while effective in decreasing viscosity, also decreases the transference number without a significant increase in conductivity. Given these factors, we conclude that salt-in-solvent and salt-solvate electrolytes are still superior to HCEs and LHCEs from a bulk transport perspective. We note that despite worse overall bulk transport, high concentration electrolytes can improve interfacial transport and stability and, therefore, could still be preferable to salt-in-solvent electrolytes for high-rate applications.

## Supporting Information Available

Experimental materials and methods (section S1, Figures S1–S3, Tables S1 and S2), additional characterization data (section S1), and additional complementary analysis of transport properties (sections S2–S5, Figures S4–S7) (PDF)

## References

[ref1] Suo L.; Hu Y.-S.; Li H.; Armand M.; Chen L. A new class of solvent-in-salt electrolyte for high-energy rechargeable metallic lithium batteries. Nat. Commun. 2013, 4, 1481 10.1038/ncomms2513. 23403582

[ref2] Yamada Y.; Yamada A. Superconcentrated electrolytes for lithium batteries. J. Electrochem. Soc. 2015, 162, A2406 10.1149/2.0041514jes.

[ref3] Zeng Z.; Murugesan V.; Han K. S.; Jiang X.; Cao Y.; Xiao L.; Ai X.; Yang H.; Zhang J.-G.; Sushko M. L.; Liu J. Non-flammable electrolytes with high salt-to-solvent ratios for Li-ion and Li-metal batteries. Nat. Energy 2018, 3, 674–681. 10.1038/s41560-018-0196-y.

[ref4] Yamada Y.; Wang J.; Ko S.; Watanabe E.; Yamada A. Advances and issues in developing salt-concentrated battery electrolytes. Nature Energy 2019, 4, 269–280. 10.1038/s41560-019-0336-z.

[ref5] Borodin O.; Self J.; Persson K. A.; Wang C.; Xu K. Uncharted waters: super-concentrated electrolytes. Joule 2020, 4, 69–100. 10.1016/j.joule.2019.12.007.

[ref6] Chen S.; Zheng J.; Mei D.; Han K. S.; Engelhard M. H.; Zhao W.; Xu W.; Liu J.; Zhang J.-G. High-voltage lithium-metal batteries enabled by localized high-concentration electrolytes. Adv. Mater. 2018, 30, 1706102 10.1002/adma.201706102. 29575163

[ref7] Cao X.; Jia H.; Xu W.; Zhang J.-G. Localized high-concentration electrolytes for lithium batteries. J. Electrochem. Soc. 2021, 168, 010522 10.1149/1945-7111/abd60e.

[ref8] Jia H.; Kim J.-M.; Gao P.; Xu Y.; Engelhard M. H.; Matthews B. E.; Wang C.; Xu W. A Systematic Study on the Effects of Solvating Solvents and Additives in Localized High-Concentration Electrolytes over Electrochemical Performance of Lithium-Ion Batteries. Angew. Chem. 2023, 135, e202218005 10.1002/ange.202218005. 36859655

[ref9] Jiang G.; Li F.; Wang H.; Wu M.; Qi S.; Liu X.; Yang S.; Ma J. Perspective on high-concentration electrolytes for lithium metal batteries. Small Struct. 2021, 2, 2000122 10.1002/sstr.202000122.

[ref10] Ugata Y.; Shigenobu K.; Tatara R.; Ueno K.; Watanabe M.; Dokko K. Solvate electrolytes for Li and Na batteries: structures, transport properties, and electrochemistry. Phys. Chem. Chem. Phys. 2021, 23, 21419–21436. 10.1039/D1CP02946K. 34550122

[ref11] Perez Beltran S.; Cao X.; Zhang J.-G.; Balbuena P. B. Localized high concentration electrolytes for high voltage lithium–metal batteries: correlation between the electrolyte composition and its reductive/oxidative stability. Chem. Mater. 2020, 32, 5973–5984. 10.1021/acs.chemmater.0c00987.

[ref12] Borodin O.; Suo L.; Gobet M.; Ren X.; Wang F.; Faraone A.; Peng J.; Olguin M.; Schroeder M.; Ding M. S.; et al. Liquid structure with nano-heterogeneity promotes cationic transport in concentrated electrolytes. ACS Nano 2017, 11, 10462–10471. 10.1021/acsnano.7b05664. 29016112

[ref13] Okoshi M.; Chou C.-P.; Nakai H. Theoretical analysis of carrier ion diffusion in superconcentrated electrolyte solutions for sodium-ion batteries. J. Phys. Chem. B 2018, 122, 2600–2609. 10.1021/acs.jpcb.7b10589. 29433319

[ref14] Yamada Y.; Furukawa K.; Sodeyama K.; Kikuchi K.; Yaegashi M.; Tateyama Y.; Yamada A. Unusual stability of acetonitrile-based superconcentrated electrolytes for fast-charging lithium-ion batteries. J. Am. Chem. Soc. 2014, 136, 5039–5046. 10.1021/ja412807w. 24654781

[ref15] Wang A. A.; Gunnarsdóttir A. B.; Fawdon J.; Pasta M.; Grey C. P.; Monroe C. W. Potentiometric MRI of a Superconcentrated Lithium Electrolyte: Testing the Irreversible Thermodynamics Approach. ACS Energy Letters 2021, 6, 3086–3095. 10.1021/acsenergylett.1c01213. 34541321 PMC8438662

[ref16] Wang A. A.; Hou T.; Karanjavala M.; Monroe C. W. Shifting-reference concentration cells to refine composition-dependent transport characterization of binary lithium-ion electrolytes. Electrochim. Acta 2020, 358, 136688 10.1016/j.electacta.2020.136688.

[ref17] Hou T.; Monroe C. W. Composition-dependent thermodynamic and mass-transport characterization of lithium hexafluorophosphate in propylene carbonate. Electrochim. Acta 2020, 332, 135085 10.1016/j.electacta.2019.135085.

[ref18] Liu J.; Monroe C. W. Solute-volume effects in electrolyte transport. Electrochim. Acta 2014, 135, 447–460. 10.1016/j.electacta.2014.05.009.

[ref19] Watanabe Y.; Ugata Y.; Ueno K.; Watanabe M.; Dokko K. Does Li-ion transport occur rapidly in localized high-concentration electrolytes?. Phys. Chem. Chem. Phys. 2023, 25, 3092–3099. 10.1039/D2CP05319E. 36621826

[ref20] Rushing J. C.; Stern C. M.; Elgrishi N.; Kuroda D. G. Tale of a “Non-interacting” Additive in a Lithium-Ion Electrolyte: Effect on Ionic Speciation and Electrochemical Properties. J. Phys. Chem. C 2022, 126, 2141–2150. 10.1021/acs.jpcc.1c09193. PMC8820140 35145574

[ref21] Einstein A. Investigations on the Theory of the Brownian Movement; Dover Books on Physics Series; Dover Publications, 1956.

[ref22] Walden P. Über organische lösungs- und ionisierungsmittel: III. Teil: Innere reibung und deren zusammenhang mit dem leitvermögen. Z. Phys. Chem. 1906, 55U, 207–249. 10.1515/zpch-1906-5511.

[ref23] Zhang X.; Zou L.; Xu Y.; Cao X.; Engelhard M. H.; Matthews B. E.; Zhong L.; Wu H.; Jia H.; Ren X.; et al. Advanced electrolytes for fast-charging high-voltage lithium-ion batteries in wide-temperature range. Adv. Energy Mater. 2020, 10, 2000368 10.1002/aenm.202000368.

[ref24] Ren X.; Chen S.; Lee H.; Mei D.; Engelhard M. H.; Burton S. D.; Zhao W.; Zheng J.; Li Q.; Ding M. S.; et al. Localized high-concentration sulfone electrolytes for high-efficiency lithium-metal batteries. Chem 2018, 4, 1877–1892. 10.1016/j.chempr.2018.05.002.

[ref25] van Ekeren W. W.; Albuquerque M.; Ek G.; Mogensen R.; Brant W. R.; Costa L. T.; Brandell D.; Younesi R. A comparative analysis of the influence of hydrofluoroethers as diluents on solvation structure and electrochemical performance in non-flammable electrolytes. Journal of Materials Chemistry A 2023, 11, 4111–4125. 10.1039/D2TA08404J.

[ref26] Wohlfarth C. Static Dielectric Constants of Pure Liquids and Binary Liquid Mixtures: Supplement to Vol. 4/17; Landolt-Bornstein: Numerical Data and Functional Relationships in Science and Technology—New Series; Springer, 2015.

[ref27] Hartley G. XLI. Theory of the velocity of diffusion of strong electrolytes in dilute solution. London, Edinburgh, Dublin Philos. Mag. J. Sci. 1931, 12, 473–488. 10.1080/14786443109461823.

[ref28] Sachar H. S.; Marioni N.; Zofchak E. S.; Ganesan V. Impact of Ionic Correlations on Selective Salt Transport in Ligand-Functionalized Polymer Membranes. Macromolecules 2023, 56, 2194–2208. 10.1021/acs.macromol.2c02380.

[ref29] Bergstrom H. K.; Fong K. D.; Halat D. M.; Karouta C. A.; Celik H. C.; Reimer J. A.; McCloskey B. D. Ion correlation and negative lithium transference in polyelectrolyte solutions. Chemical Science 2023, 14, 6546–6557. 10.1039/D3SC01224G. 37350831 PMC10283486

[ref30] Fong K. D.; Bergstrom H. K.; McCloskey B. D.; Mandadapu K. K. Transport phenomena in electrolyte solutions: Nonequilibrium thermodynamics and statistical mechanics. AIChE J. 2020, 66, e17091 10.1002/aic.17091.

[ref31] Kilchert F.; Lorenz M.; Schammer M.; Nürnberg P.; Schönhoff M.; Latz A.; Horstmann B. A Volume-based Description of Transport in Incompressible Liquid Electrolytes and its Application to Ionic Liquids. Phys. Chem. Chem. Phys. 2023, 25, 25965 10.1039/D2CP04423D. 37646123

[ref32] Lorenz M.; Kilchert F.; Nurnberg P.; Schammer M.; Latz A.; Horstmann B.; Schonhoff M. Local volume conservation in concentrated electrolytes is governing charge transport in electric fields. J. Phys. Chem. Lett. 2022, 13, 8761–8767. 10.1021/acs.jpclett.2c02398. 36102654

[ref33] Timachova K.; Newman J.; Balsara N. P. Theoretical interpretation of ion velocities in concentrated electrolytes measured by electrophoretic NMR. J. Electrochem. Soc. 2019, 166, A264 10.1149/2.0591902jes.

[ref34] Fong K. D.; Self J.; McCloskey B. D.; Persson K. A. Onsager transport coefficients and transference numbers in polyelectrolyte solutions and polymerized ionic liquids. Macromolecules 2020, 53, 9503–9512. 10.1021/acs.macromol.0c02001.

[ref35] Shigenobu K.; Shibata M.; Dokko K.; Watanabe M.; Fujii K.; Ueno K. Anion effects on Li ion transference number and dynamic ion correlations in glyme–Li salt equimolar mixtures. Phys. Chem. Chem. Phys. 2021, 23, 2622–2629. 10.1039/D0CP06381A. 33475115

[ref36] Shigenobu K.; Dokko K.; Watanabe M.; Ueno K. Solvent effects on Li ion transference number and dynamic ion correlations in glyme-and sulfolane-based molten Li salt solvates. Phys. Chem. Chem. Phys. 2020, 22, 15214–15221. 10.1039/D0CP02181D. 32598420

[ref37] Hertz H. Velocity Correlations in Aqueous Electrolyte Solutions from Diffusion, Conductance, and Transference Data. Part 1, Theory. Berichte der Bunsengesellschaft für physikalische Chemie 1977, 81, 656–664. 10.1002/bbpc.19770810707.

[ref38] Friedman H. L.; Mills R. Hydrodynamic approximation for distinct diffusion coefficients. Journal of solution chemistry 1986, 15, 69–80. 10.1007/BF00646311.

[ref39] Dong D.; Sälzer F.; Roling B.; Bedrov D. How efficient is Li+ ion transport in solvate ionic liquids under anion-blocking conditions in a battery?. Phys. Chem. Chem. Phys. 2018, 20, 29174–29183. 10.1039/C8CP06214E. 30426990

[ref40] Kashyap H. K.; Annapureddy H. V.; Raineri F. O.; Margulis C. J. How is charge transport different in ionic liquids and electrolyte solutions?. J. Phys. Chem. B 2011, 115, 13212–13221. 10.1021/jp204182c. 22022889

[ref41] Sudoh T.; Ikeda S.; Shigenobu K.; Tsuzuki S.; Dokko K.; Watanabe M.; Shinoda W.; Ueno K. Li-Ion Transport and Solution Structure in Sulfolane-Based Localized High-Concentration Electrolytes. J. Phys. Chem. C 2023, 127, 12295–12303. 10.1021/acs.jpcc.3c02112.

[ref42] Grundy L. S.; Shah D. B.; Nguyen H. Q.; Diederichsen K. M.; Celik H.; DeSimone J. M.; McCloskey B. D.; Balsara N. P. Impact of frictional interactions on conductivity, diffusion, and transference number in ether-and perfluoroether-based electrolytes. J. Electrochem. Soc. 2020, 167, 120540 10.1149/1945-7111/abb34e.

