# Supplementary material for: Ion Transport in (Localized) High Concentration Electrolytes for Li-Based Batteries
Source: ACS Energy Lett. 2024 Jan 5;9(2):373–80. doi: 10.1021/acsenergylett.3c01662 (PMC10863389; doi:10.1021/acsenergylett.3c01662)
Supplement: Supplementary file 1 — nz3c01662_si_001.pdf [file nz3c01662_si_001.pdf]

Supporting Information: Ion Transport in (Localized) High Concentration for Li-based Batteries

Helen K. Bergstrom,[1,2‡] Bryan D. McCloskey[1,2§]

[1] Department of Chemical & Biomolecular Engineering, University of California, Berkeley, CA 94720, USA

[2] Energy Storage and Distributed Resources Division, Lawrence Berkeley National Laboratory, Berkeley, CA 94720, USA

‡ helen_bergstrom@berkeley.edu

§ bmcclosk@berkeley.edu

S1 Experimental Methods

S1.1 Materials

Battery grade DMC and LiFSI were purchased from Gotion Inc. and directly transferred under inert atmosphere to an argon glovebox (MTI) kept below 5 ppm water and oxygen. High purity TTE was purchased from Synquest and directly transferred under argon to the glovebox. Poly(vinylidene fluoride) (PVDF) was purchased from Sigma Aldrich (average MW ∼534,000) and dried at 105°C under vacuum for 12 hours prior to transfer to the glovebox. Lithium metal foil (0.75mm thick) was obtained from MTI Corporation and lithium metal wire (3.2mm diameter) was obtained from Alfa-Aesar. All lithium was brushed with nylon bristles to remove the native surface layer prior to use.

S1.2 Electrolyte Preparation & Physical Characterization

All electrolyte solutions were prepared inside the glovebox using a moles of Li^+ per kg DMC basis. For electrophoretic NMR measurements of the 1.1m and 2.8m LiFSI in DMC electrolytes PVDF was added (3.4 wt.% and 3.9 wt.% respectively) as a gelling agent to suppress convection. Gelled samples were used only for electrophoretic NMR measurements. Due to their high viscosity, HCEs with concentrations $\geq$ 5.55m and LHCEs were not gelled with PVDF for eNMR measurements. For electrolytes gelled with PVDF, first the LiFSI was fully dissolved in DMC prior to addition of PVDF. The solution was then rapidly heated to 120°C while stirring until a clear solution was obtained before the sample was cooled back to room temperature. To prevent LiFSI degradation, high temperature exposure was limited. For LHCEs, the LiFSI was fully dissolved in DMC prior to addition of TTE. We noted that the addition of TTE negatively impacted the solubility of LiFSI in DMC. The solubility limit at 30° given the 2:1 molar ratio of DMC to TTE was approximately 9.25 mol/kg DMC (1:1.20:0.60 mols Li:DMC:TTE). Solution densities were measured in an Anton Paar DMA 4101 oscillating U-tube density meter at 30°C inside an argon glovebox. Each density measurement was performed in triplicate. Viscosity measurements were performed in triplicate in an electromagnetically spinning viscometer (EMS-1000s, Kyoto Electronics) spinning at 1000 rotations per minute at 30°C. Samples were sealed inside air-tight vials inside the glovebox before transfer to the viscometer to ensure no moisture or air contamination. In order to avoid ambiguity in solution concentration definitions, we also report the

particle fraction of salt in each solution (y) according to

$$y = \frac{N_s}{\nu N_s + N_0 + N_D} = \frac{\frac{M_0}{M_s}\omega_s}{1 + \left(\frac{M_0}{M_D} - 1\right)\omega_D + \left(\frac{\nu M_0}{M_s} - 1\right)\omega_s} \tag{S1}$$

where N_i is number of mols of salt (s), solvent (0) and diluent (D) respectively, M_i is the molar mass of species 'i', ω_i is the mass fraction of species 'i' and ν is the salt stoichiometric coefficient (2 for LiFSI). The salt particle fraction is convenient because y is independent of temperature and pressure and unlike molality does not require a somewhat arbitrary definition of the solvent in mixed solvent systems or micro-phase separated systems (such as the case of localized high concentrated electrolytes).[1]

S1.3 Separator Characterization

In order to obtain accurate bulk transport properties for liquid electrolytes measured within a porous separator, it is necessary to know the separator tortuosity (τ) and porosity (ϵ) or conducting fraction (ϕ_c). Glass fiber separators were chosen over common polypropylene separators (Celgard) due to wetting issues at high concentration. The porosity and conducting fraction of Whatman QMA disks were determined using methods described in ref[2] assuming a density of quartz fiber of 2.2 g/cm^3. QMA disks were dried for at least 24 hours at 120°C prior to use. Electrolyte uptake was determined with pure DMC. Tortuosity was measured by comparing the bulk conductivity to conductivity within the QMA separator (κ_s) for aqueous conductivity standards (Mettler Toledo). Conductivity within the QMA separator was measured against blocking stainless steel electrodes in coin cells using AC impedance spectroscopy on a Bio-Logic VMP3 potentiostat in the frequency range from 1 MHz to 100 mHz with a 5 mV AC amplitude. QMA can compress significantly therefore to keep cell thickness constant, QMA disks were set inside a 0.762mm thick PEEK washer prior to loading with 150μL of electrolyte. Impedance data was tested for linearity using a Kramers-Kronig analysis and fit to R-RQ equivalent circuit using the open-source Py-EIS package for Python.[3] Conductivity was calculated according to

$$\kappa_s = \frac{l}{R_{hf} A} \tag{S2}$$

where l is the inter-electrode distance, R_{hf} is the high frequency resistance, and A is the geometric electrode area. Tortuosity was then calculated according to

$$\tau = \phi_c \frac{\kappa}{\kappa_s}. \tag{S3}$$

Values for the measured physical properties of our Whatman QMA separators are presented in Table S1.

Table S1: Measured physical properties of Whatman QMA glass fiber separator

Property	Measured Value
Porosity	0.917 ± 0.0004
Conducting Fraction	0.944 ± 0.0005
Tortuosity	1.27 ± 0.10

S1.4 Conductivity

Liquid electrolyte conductivity (κ) was measured using a Mettler Toledo InLab 751-4mm conductivity probe with blocking platinum electrodes inside the glovebox. The conductivity probe was calibrated using 84 μS/cm, 1413 μS/cm, and 12.88 mS/cm aqueous standards (Mettler Toledo) prior to bringing it inside the glovebox. Samples were maintained at 30±°C using a dry block and solution temperatures were verified using

the probe's internal sensor. A 5% error is estimated for probe measurements based on replicate measurements. For gelled samples, accurate measurement with the conductivity probe at 30°C was not possible. Gelled electrolyte conductivity was measured inside a fused electrophoretic NMR cell (P&L Scientific) using AC impedance spectroscopy (Bio-Logic SP-300 potentiostat) in the frequency range of 1MHz to 100 mHz with a 10mV AC amplitude. The cell constant of the NMR cell was measured using 84 μS/cm, 1413 μS/cm, and 12.88 mS/cm aqueous standards. Impedance data was tested for linearity using a Kramers-Kronig analysis and fit to RQ equivalent circuit using the open-source Py-EIS package for Python.[3]

S1.5 Restricted Diffusion

Restricted diffusion measurements were performed inside lithium symmetric coin cells. Two QMA disks impregnated with 150μL of electrolyte were set inside a 0.762mm thick PEEK washer and sandwiched between 15 mm brushed lithium electrodes inside a CR2032 coin cell (Hohsen Corporation). Three replicate cells were made for each concentration. Cells were run inside an environmental chamber (Thermotron Inc.) maintained at 30°C and allowed to equilibrate at open circuit potential for 12 hours prior to testing. Cells were polarized at 10 mV for twelve hours to allow concentration gradients to build before allowing the cell to relax at open circuit for twelve hours with potential recorded every 0.5 seconds. To minimize the influence of interfacial noise at long times (low voltages), we logarithmically downsampled the data prior to fitting.[4] The effective total salt diffusion coefficient ($D_{\pm}^{\text{eff}}$) was obtained by fitting the voltage relaxation to Equation S4 where l is the electrode separation distance.[4–6] The electrolyte total diffusion coefficient ($D_{\pm}$) is obtained by multiplying $D_{\pm}^{\text{eff}}$ by the separator tortuosity.

$$U(t) = \alpha \exp\left(\frac{-\pi^2 D_{\pm}^{\text{eff}}}{l^2} t\right) \tag{S4}$$

S1.6 Concentration Cells

Concentration cells were constructed inside a custom fabricated low-volume glass U-cell with a P4 glass frit (Adams & Chittenden). Concentration cell measurements were performed inside the argon glovebox with cell temperature maintained using a dry block with each U-cell equilibrated at 30°C prior to electrolyte addition. The change in the liquid junction potential across the cell with varied concentration is a function of both the transference number and the electrolyte solution activity. Because we expect t_+ to vary significantly across concentration, we chose to adopt the "shifting-reference" concentration cell method introduced by Wang et al.[7] For each reference concentration, five concentration combinations were tested with the test concentrations selected to produce reliably measurable liquid junction potentials ~5 - 10 mV without changing the salt:solvent ratio significantly. For the LHCE the upper concentrations were determined by the salt solubility limit. U-cells were constructed using 1mL of electrolyte added on each side of the glass frit before brushed lithium metal wire electrodes were immersed in the solution on each side. The open-circuit potential, U(t), was recorded over the course of 1 hour. Each concentration combination was performed in triplicate. While 3rd order polynomial fits of concentration cell potential vs. log of salt concentration are often used in the literature, there is no physical reason for this choice, and without sufficient data there can be significant over-fitting. Here, we choose the most parsimonious fit of $U(m - m_{\text{ref}})$ vs. $\log(m/m_{\text{ref}})$ which in most cases was a linear fit (see Fig. S1). The derivative of concentration cell potential with respect to concentration is then related to the thermodynamic factor (χ) according to

$$\chi(m_{\text{ref}}) = \frac{\partial U}{\partial \ln m}\bigg|_{m=m_{\text{ref}}} \frac{z_+ \nu_+ F}{\nu RT} \frac{1}{1 - t_+^0(m_{\text{ref}})}. \tag{S5}$$

S1.7 Pulsed Field Gradient and Electrophoretic NMR

NMR samples were prepared inside the glovebox and loaded into specialized eNMR sample cells (P&L Scientific) consisting of a 5 mm glass tube with palladium electrodes with an approximate inter-electrode

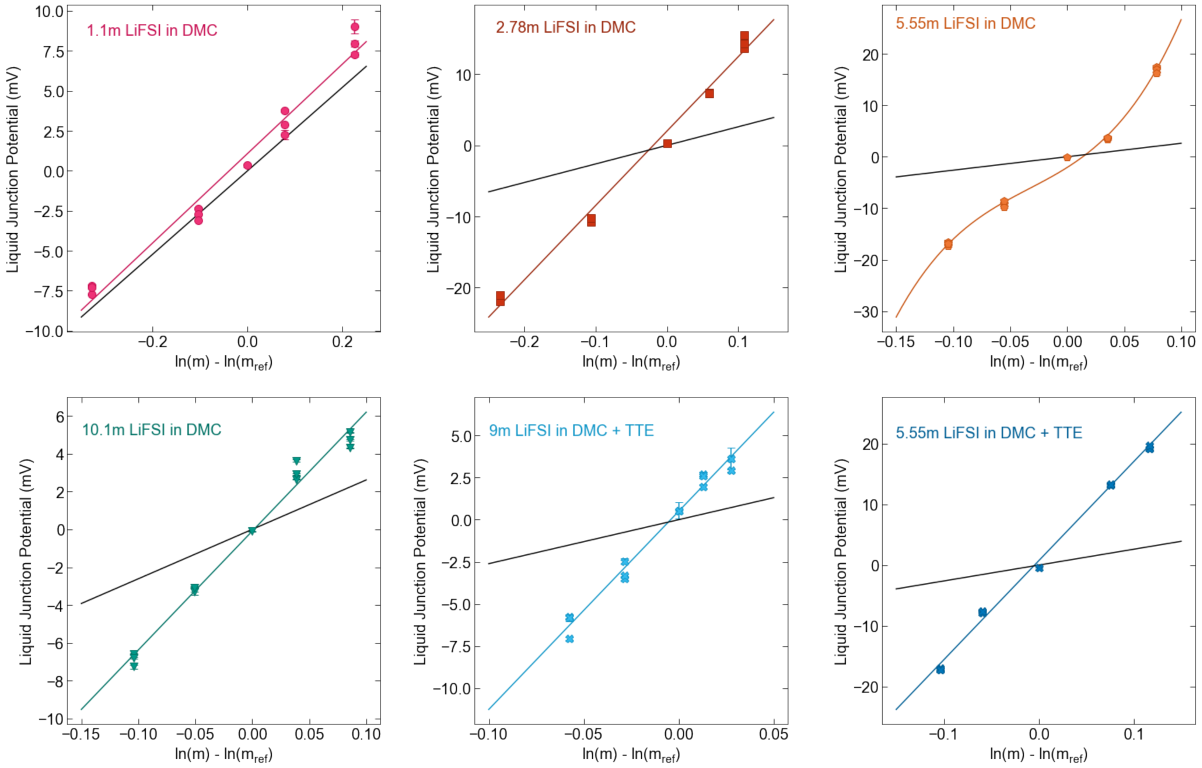

Figure S1: Concentration cell potential vs. log of molality difference. Here the black line represents the concentration cell potential calculated using the Nernst equation (assuming ideal solution behavior). Note: star symbols denote the LHCE of LiFSI in DMC and TTE. Molalities are reported with respect to DMC weight not total solvent (DMC + TTE) weight.

distance of 3.35 cm, and an air-tight teflon cap.[8] Exact inter-electrode distances were calibrated by measuring the mobility of a 10mM tetramethylammonium bromide solution in deuterated water and comparing to literature values.[8,9] Pulsed field gradient (PFG) NMR and eNMR measurement were performed at a field strength of 9.4T on a Bruker NEO 400 MHz spectrometer fitted with a 5 mm water-cooled double resonance broadband diffusion (diffBB) probe equipped with z-axis gradient capabilities up to 17T/m and a variable temperature unit that was maintained at 30°C throughout measurements. The 90° pulse time for each peak of interest was measured. Separate ^{19}F experiments were performed for TTE and FSI peaks due to significant difference in each species ^{19}F carrier frequency. For PFG measurements, a double stimulated echo bipolar gradient pulse sequence (Bruker pulse sequence diffDSTEAV3) with sin-bell magnetic field gradient pulses (SIN.100) was used in order to eliminate convection-based artifacts for all 3 measured nuclei (^{1}H, ^{19}F, ^{7}Li).[10] Eight dummy gradient pulses and sixteen dummy scans were applied at the beginning of each program prior to spectral acquisition to warm up the gradient amps and ensure sample equilibration. For each peak of interest, 16 linearly spaced gradient steps were acquired with the gradient parameters optimized such that the signal attenuates over at least one order of magnitude. PFG data was fit to the Stejskal-Tanner equation

$$\frac{I}{I_0} = \exp - \left[D_i^{\mathrm{self}} \gamma^2 g^2 \delta^2 \left(\Delta - \frac{5\delta}{8} - \frac{\tau_1 + \tau_2}{2} \right) \right] \tag{S6}$$

where D_i^{self} is the self diffusion coefficient of species i, γ is the gyromagnetic ratio, g is the gradient strength including a correction for the sin-bell shape factor, δ is the gradient pulse duration, Δ is the drift delay, and τ_1 and τ_2 are the gradient recovery delays.[11]

For eNMR experiments, electric field pulses were applied with a P&L eNMR 1000 electrophoretic high-voltage amplifier unit (P&L Scientific Instrument Services).[8,9] eNMR amplifier pulses were controlled by incoming trigger pulses from the Bruker spectrometers to synchronize the electric field pulses with radio frequency (rf) and magnetic field gradient of the eNMR pulse program. Noise from rf pulses was suppressed using a two-stage electronic filter assembly - the first grounded on the NMR preamplifier and the second embedded in the eNMR cell holder provided by P&L. A convection-compensated double stimulated echo eNMR pulse sequence[12,13] was used with bipolar electric field pulses lasting 50 ms each[14,15] to reduce error induced by possible convection, electro-osmotic flow, and bubble formation. eNMR measurements were performed with voltage-controlled electric field pulses with the applied voltage range chosen on a per-sample basis. The lower end of the voltage range was selected such that a phase shift was discernible, $\sim 1°$, while the upper voltage range was selected as the highest voltage before significant signal attenuation due to convection was observed. In order to eliminate any systematic spurious phase shifts that do arise from artifacts, we duplicated each experiment with positive and negative gradient encoding which should result in equal magnitude, but opposite sign phase shifts as a function of applied voltage.[15,16] Due to the short-lived nature of electric-field pulses and blocking nature of palladium electrodes, eNMR measurements should not be affected by solution-volume change driven flow (e.g. excluded volume effects or Faradaic convection),[17] or bulk diffusion. Due to the high conductivity and low viscosity of the 1.1m and 2.78m samples, it was not possible to find conditions that did not suffer from significant joule-heating related convective and electro-osmotic artifacts. To overcome this, we added a small ($\sim$ 3-4) weight percent PVDF to gel these samples[18] which eliminated most convective artifacts while proportionally lowering the motion of all species by $\sim$10%. Self diffusion coefficients of the pristine and gelled solutions were compared to ensure that gelling affected all measured species equally. Gelled samples were only used to obtain electrophoretic mobilities and transference number, and all other data presented herein for 1.1m and 2.78m samples are from measurements of pure liquid samples.

eNMR experiments measure ion drift velocities in an electric field which manifests as a phase angle shift in the NMR signal. The phase shift $(\Phi - \Phi_0)$ is directly related to the drift velocity, $\boldsymbol{v}$ and magnetic field gradient parameters according to

$$(\Phi - \Phi_0) = \gamma \delta \Delta g \boldsymbol{v}. \tag{S7}$$

The electrophoretic mobility of a species i (μ_i) can then be related to the drift velocity at a given the electric field (E) according to

$$\mu_i = \frac{\boldsymbol{v}_i - \boldsymbol{v}_{ref}}{E}. \tag{S8}$$

To systematically determine the phase of each spectra, we used a modified version of the open source python package *eNMRpy* to perform phase-sensitive spectral deconvolution assuming Lorentzian line shapes.[16,19,20] For each peak fit, we used a matched filter condition such that line broadening matched the natural linewidth of the peak of interest. In order to ensure that our measurements were free of major artifacts, we compared conductivity of our solution obtained by impedance spectroscopy on each sample to those calculated using our measured ion mobilities. Representative values for gradient and voltage parameters used in electrophoretic (eNMR) experiments are listed in Table S2. Exemplary phase shift data vs. $g \cdot V \cdot \delta \cdot L^{-1}$ for each nucleus is presented in Figs. S2, S3 where g is the gradient strength in Tesla per meter, V is the applied voltage in volts, δ is the drift time in seconds, and L is the electrode separation distance in meters.

Velocities from eNMR are all with reference to the stationary NMR probe ($\boldsymbol{v}_{ref} = 0$). We can switch to a solvent velocity reference frame denoted by a superscript '0' by subtracting the solvent velocity from other species velocities. Here we take the solvent to be DMC for both HCEs and LHCEs. To convert to a center of mass reference frame denoted by a superscript 'COM', we can calculate the center of mass velocity according to

$$\boldsymbol{v}^{\mathrm{COM}} = \sum \omega_i \boldsymbol{v}_i \tag{S9}$$

where ω_i is the mass fraction of species i in the solution.[4]

Table S2: Representative eNMR parameter values

Regime	Nucleus	Gradient Strength (T/m)	Applied Voltage (V)
1-5.5m	^{7}Li	0.8-3	0-150
	^{19}F	0.3-1.1	0-150
	^{1}H	0.25-0.9	0-150
9-10m	^{7}Li	4-7	0-300
	^{19}F	0.8-2.75	0-300
	^{1}H	1.25-2.5	0-300

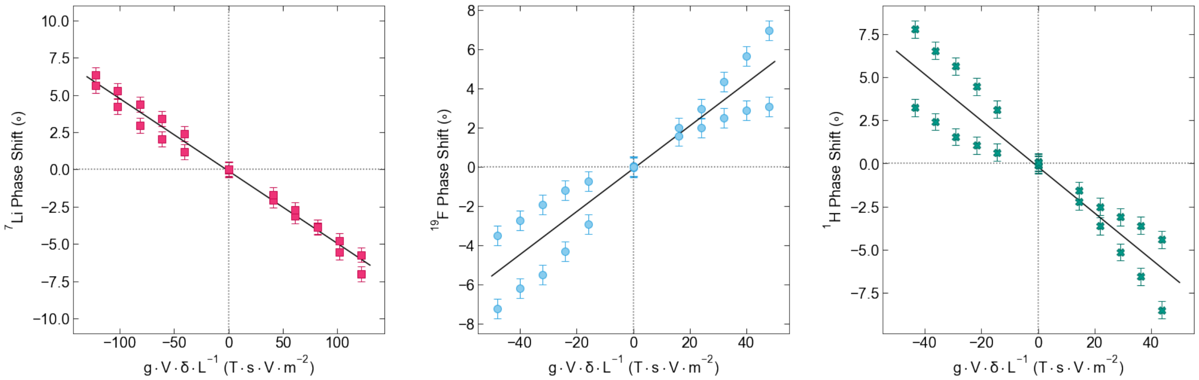

Figure S2: Phase shift (degrees) vs. $g \cdot V \cdot \delta \cdot L^{-1}$ $(T \cdot s \cdot V \cdot m^{-2})$ for three studied nuclei of 1:1.1 molar ratio of LiFSI:DMC (10.1m) high concentration electrolyte. ^{7}Li points correspond to lithium ions in solution and associated with FSI, ^{19}F points correspond to the FSI anion, and ^{1}H points correspond to DMC.

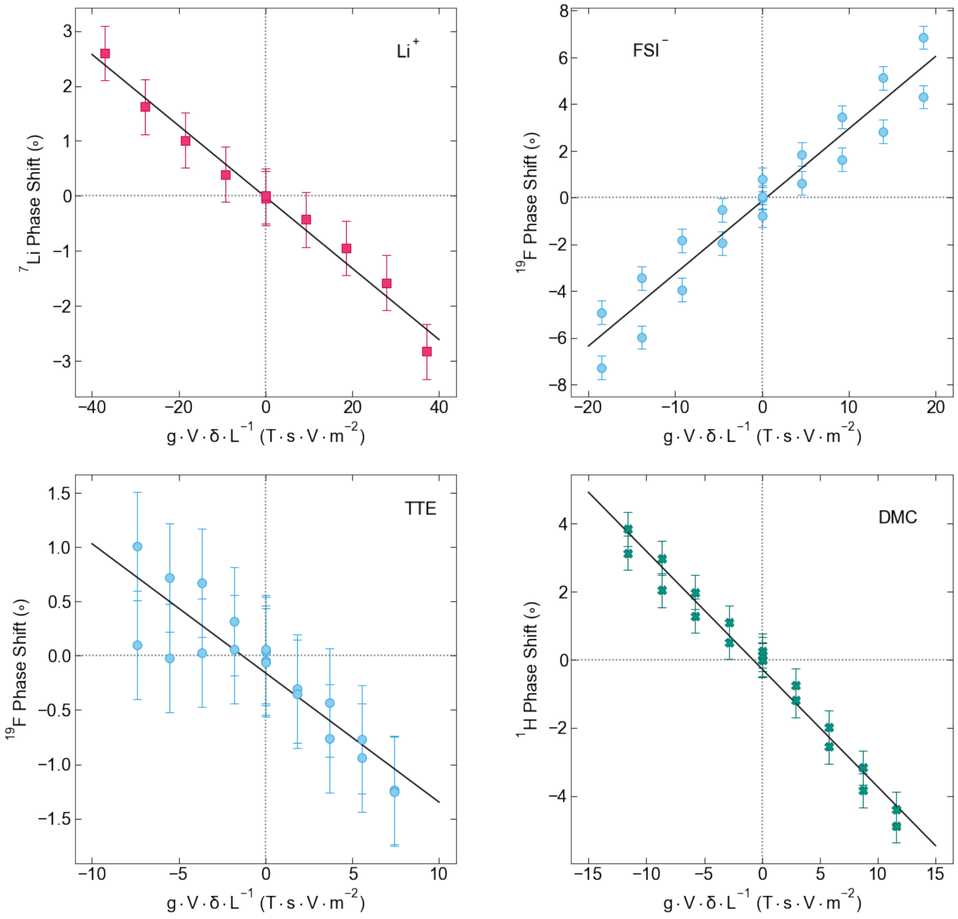

Figure S3: Phase shift (degrees) vs. $g \cdot V \cdot \delta \cdot L^{-1}$ $(T \cdot s \cdot V \cdot m^{-2})$ for three studied nuclei of 1:1.23:0.62 molar ratio of LiFSI:DMC:TTE (9m) localized high concentration electrolyte. ^{7}Li points correspond to lithium ions in solution and associated with FSI, ^{19}F points in the upper right panel correspond to the FSI anion, ^{19}F points in the lower left panel correspond to the TTE diluent, and ^{1}H points correspond to DMC.

S2 Effect of Solution Activity on the Total Salt Diffusion Coefficient

In order to understand if changes in the solution activity coefficient are responsible for differences between the measured total salt diffusion coefficient ($D_\pm$) and the ideal salt diffusion coefficient obtained by the Nernst-Heartly equation ($D_\pm^{\text{ideal}}$), we can multiply $D_\pm^{\text{ideal}}$ by the thermodynamic factor (χ). The quantity $D_\pm^{\text{ideal}} \cdot \chi$ captures the "ideal" diffusion coefficient with respect to the gradient in solution activity instead of gradients in solution concentration. $D_\pm$, $D_\pm^{\text{ideal}}$, and $D_\pm^{\text{ideal}} \cdot \chi$ are plotted versus solution concentration in Figure S4. We observe for all electrolytes except the 10.1m (1.1:1 DMC:LiFSI) HCE that difference the thermodynamic factor is responsible for differences in $D_\pm$ and $D_\pm^{\text{ideal}}$.

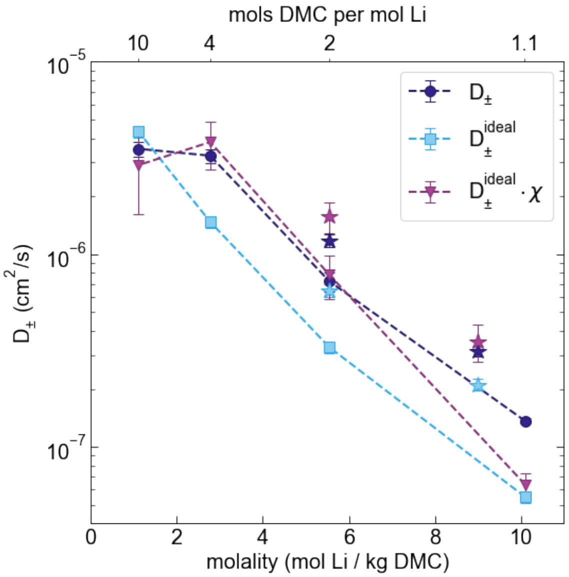

Figure S4: Total salt diffusion coefficients (cm^2/s) vs. molality (mol Li^+/kg DMC) and DMC:Li molar ratio as measured using restricted diffusion and as calculated from PFG NMR assuming ideal solution behavior. Note: star symbols denote LHCE systems composed of LiFSI in DMC and TTE. Molalities are reported with respect to DMC weight not total solvent (DMC + TTE) weight.

S3 Effective Ion Charge

We can calculate an effective ion charge (z_i^{eff})[21–23] by performing a force balance between the Coulombic forces under an electric field and the hydrodynamic friction forces on ions during an eNMR experiment, according to

$$z_i^{\text{eff}} = \frac{\mu_i RT}{F D_i^{\text{self}}} \tag{S10}$$

.

We see the cation effective charge increase with increasing molality when from a static picture of ion-pairing we would expect more ion pairing and therefore lower effective charge at high concentration (see Fig. S5). This indicates that a static picture of ion-pairing is not appropriate for these systems and that at high concentration effective charge is higher due to shorter lived ion pairs (fast ligand exchange). We observe for the LHCE the effective charge of both the Li^+ and FSI^- ions are reduced, again indicating that the diluent addition impacts ion-solvation dynamics.

S4 Lithium Transference Number Reference Frames

Using electrophoretic NMR we can calculate the transference number with respect to the fixed laboratory reference frame (t_+^{Lab}), center of mass reference frame (t_+^{COM}), or solvent reference frame (t_+^0). While the solvent reference frame is not particularly meaningful at high salt concentrations, it is frequently used in conventional liquid electrolyte literature and therefore is presented below in Fig. S6. A negative solvent frame transference number does not mean lithium is moving in the "wrong" direction in an electric field, simply that the solvent has a higher electrophoretic mobility than the Li-ion. As discussed in the main text, for HCEs and LHCEs the high apparent solvent mobility is a result of mass conservation in these systems.

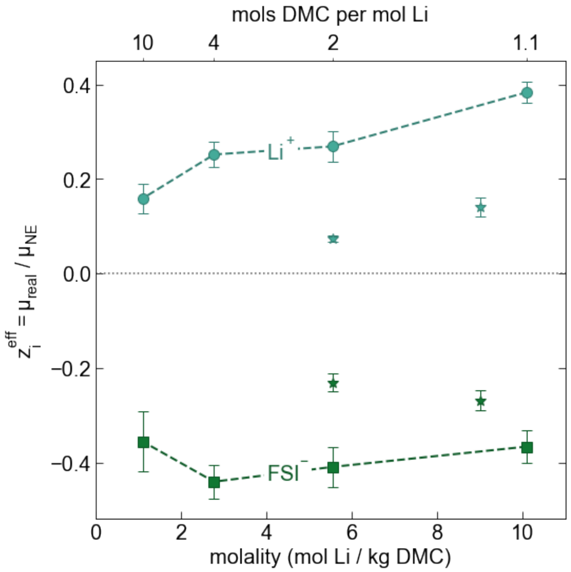

Figure S5: Effective ion charge vs. molality (mol Li^+/kg DMC) and DMC:Li molar ratio. Note: star symbols denote the LHCE of LiFSI in DMC and TTE. Molalities are reported with respect to DMC weight not total solvent (DMC + TTE) weight.

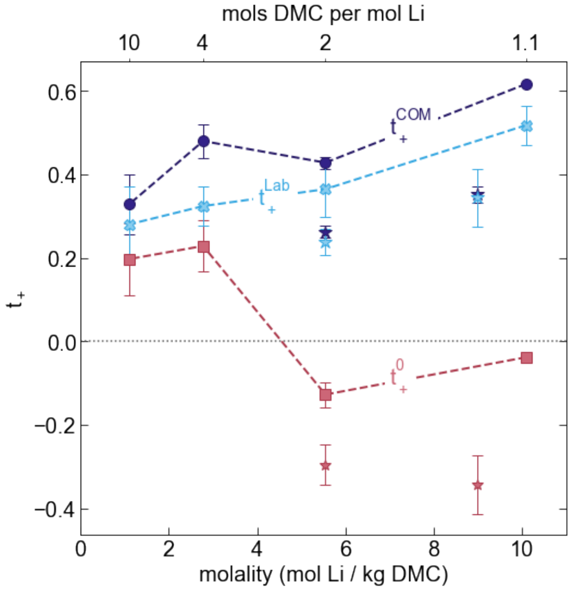

Figure S6: Li^+ transference number with respect to fixed laboratory frame (t_+^{Lab}), center of mass frame (t_+^{COM}), and solvent frame (t_+^0) vs. molality (mol Li^+/kg DMC) and DMC:Li molar ratio as measured by eNMR. Note star symbols denote LHCE systems composed of LiFSI in DMC and TTE

S5 Stefan-Maxwell Coefficients

Analogous to the Onsager transport framework, the Stefan-Maxwell transport framework can be used to understand ion-ion and ion-solvent interactions. Stefan-Maxwell diffusivites, $\mathfrak{D}_{ij}$ and their corresponding friction coefficients, K_{ij} can be calculated from experimental properties as well via an inversion process from Onsager transport coefficients.[24,25] The mutual salt diffusion coefficient based on salt chemical potential gradients, $\mathfrak{D}$ can be calculated according to

$$D_{\pm} = \frac{c_T}{c_0}\mathfrak{D}\chi \tag{S11}$$

Stefan-Maxwell diffusion coefficients are then related to transport properties through

$$\mathfrak{D}_{0+} = \frac{-z_-}{z_+ - z_-}\frac{\mathfrak{D}}{1 - t_+^0} \tag{S12}$$

$$\mathfrak{D}_{0-} = \frac{z_+}{z_+ - z_-}\frac{\mathfrak{D}}{t_+^0} \tag{S13}$$

$$\mathfrak{D}_{+-} = \left[\frac{-z_+ z_- c_T F^2}{\kappa RT} - \frac{z_+ - z_-}{z_+\nu_+}\frac{c_0 t_+^0\left(1 - t_+^0\right)}{c\mathfrak{D}}\right]^{-1} \tag{S14}$$

The corresponding friction coefficients can be calculated according to

$$K_{ij} = RT\frac{c_i c_j}{c_T}\frac{1}{\mathfrak{D}_{\mathrm{ij}}} \tag{S15}$$

Stefan-Maxwell diffusion coefficients and drag coefficients are presented below in Fig. S7.

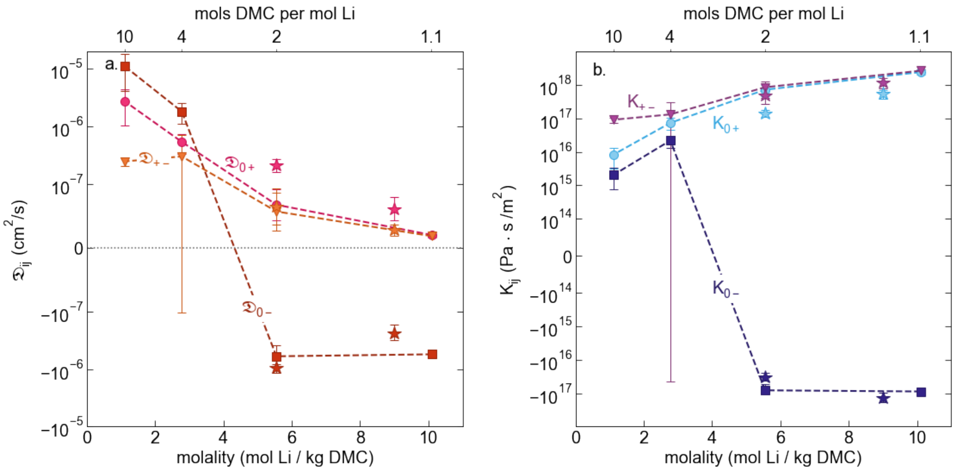

Figure S7: a)Stefan-Maxwell diffusion coefficients ($\mathfrak{D}_{ij}$) and b) Stefan-Maxwell friction coefficients (K_{ij}) vs. salt molality calculated from experimental data. Note star symbols denote LHCE systems composed of LiFSI in DMC and TTE

References

(1) Liu, J.; Monroe, C. W. On the characterization of battery electrolytes with polarization cells. *Electrochimica Acta* **2015**, *167*, 357–363.

(2) Devaux, D.; Chang, Y. H.; Villaluenga, I.; Chen, X. C.; Chintapalli, M.; DeSimone, J. M.; Balsara, N. P. Conductivity of carbonate-and perfluoropolyether-based electrolytes in porous separators. *Journal of Power Sources* **2016**, *323*, 158–165.

(3) Knudsen, K. kbknudsen/PyEIS: PyEIS: A Python-based Electrochemical Impedance Spectroscopy simulator and analyzer, version 1.0.3, 2019, `https://doi.org/10.5281/zenodo.2535951`.

(4) Bergstrom, H. K.; Fong, K. D.; McCloskey, B. D. Interfacial Effects on Transport Coefficient Measurements in Li-ion Battery Electrolytes. *Journal of The Electrochemical Society* **2021**, *168*, 060543.

(5) Harned, H. S.; French, D. M. A conductance method for the determination of the diffusion coefficients of electrolytes. *Annals of the New York Academy of Sciences* **1945**, *46*, 267–284.

(6) Newman, J.; Chapman, T. W. Restricted diffusion in binary solutions. *AIChE Journal* **1973**, *19*, 343–348.

(7) Wang, A. A.; Hou, T.; Karanjavala, M.; Monroe, C. W. Shifting-reference concentration cells to refine composition-dependent transport characterization of binary lithium-ion electrolytes. *Electrochimica Acta* **2020**, *358*, 136688.

(8) Fang, Y.; Yushmanov, P. V.; Furó, I. Improved accuracy and precision in electrophoretic NMR experiments. Current control and sample cell design. *Journal of Magnetic Resonance* **2020**, *318*, 106796.

(9) Hallberg, F.; Furó, I.; Yushmanov, P. V.; Stilbs, P. Sensitive and robust electrophoretic NMR: Instrumentation and experiments. *Journal of Magnetic Resonance* **2008**, *192*, 69–77.

(10) Jerschow, A.; Müller, N. Suppression of convection artifacts in stimulated-echo diffusion experiments. Double-stimulated-echo experiments. *Journal of Magnetic Resonance* **1997**, *125*, 372–375.

(11) Sinnaeve, D. The Stejskal–Tanner equation generalized for any gradient shape—an overview of most pulse sequences measuring free diffusion. *Concepts in Magnetic Resonance Part A* **2012**, *40*, 39–65.

(12) He, Q.; Wei, Z. Convection compensated electrophoretic NMR. *Journal of magnetic resonance* **2001**, *150*, 126–131.

(13) Zhang, Z.; Madsen, L. A. Observation of separate cation and anion electrophoretic mobilities in pure ionic liquids. *The Journal of chemical physics* **2014**, *140*, 084204.

(14) Pettersson, E.; Furo, I.; Stilbs, P. On experimental aspects of electrophoretic NMR. *Concepts in Magnetic Resonance Part A: An Educational Journal* **2004**, *22*, 61–68.

(15) Halat, D. M.; Fang, C.; Hickson, D.; Mistry, A.; Reimer, J. A.; Balsara, N. P.; Wang, R. Electric-field-induced spatially dynamic heterogeneity of solvent motion and cation transference in electrolytes. *Physical review letters* **2022**, *128*, 198002.

(16) Bergstrom, H. K.; Fong, K. D.; Halat, D. M.; Karouta, C. A.; Celik, H. C.; Reimer, J. A.; McCloskey, B. D. Ion correlation and negative lithium transference in polyelectrolyte solutions. *Chemical Science* **2023**.

(17) Liu, J.; Monroe, C. W. Solute-volume effects in electrolyte transport. *Electrochimica Acta* **2014**, *135*, 447–460.

(18) Holz, M. Electrophoretic Nmr. *Chemical Society Reviews* **1994**, *23*, 165–174.

(19) Schmidt, F.; Pugliese, A.; Santini, C. C.; Castiglione, F.; Schönhoff, M. Spectral deconvolution in electrophoretic NMR to investigate the migration of neutral molecules in electrolytes. *Magnetic Resonance in Chemistry* **2020**, *58*, 271–279.

(20) Ackermann, F. eNMRpy, version 0.0.6, 2021, `https://github.com/Flackermann/eNMRpy`.

(21) Böhme, U.; Scheler, U. Effective charge of polyelectrolytes as a function of the dielectric constant of a solution. *Journal of Colloid and interface science* **2007**, *309*, 231–235.

(22) Böhme, U.; Scheler, U. Counterion condensation and effective charge of poly (styrenesulfonate). *Advances in colloid and interface science* **2010**, *158*, 63–67.

(23) Scheler, U. In *eMagRes*; John Wiley & Sons, Ltd: 2012.

(24) Newman, J.; Thomas-Alyea, K. E., *Electrochemical Systems*, 3rd ed.; John Wiley & Sons, Inc.: 2004.

(25) Fong, K. D.; Bergstrom, H. K.; McCloskey, B. D.; Mandadapu, K. K. Transport phenomena in electrolyte solutions: Nonequilibrium thermodynamics and statistical mechanics. *AIChE Journal* **2020**, *66*, e17091.
